# Associations among COVID-19 Family Stress, Family Functioning, and Child Health-Related Quality of Life through Lifestyle Behaviors in Children

**DOI:** 10.3390/children11040483

**Published:** 2024-04-18

**Authors:** Kay W. Kim, Jan L. Wallander, Deborah Wiebe

**Affiliations:** Psychological Sciences, University of California, Merced, CA 95343, USA

**Keywords:** COVID-19 family stress, family functioning, health-related quality of life, diet

## Abstract

The COVID-19 pandemic has resulted in lasting effects on children, necessitating a thorough understanding of its impact for effective recovery planning. This study investigated the associations among COVID-19 family stress, family functioning, children’s lifestyle behaviors (i.e., healthy food intake, unhealthy food intake, physical activity, and screen time), and their health-related quality of life (HRQOL). Data from a 2022 survey of parents with children aged 5 to 12 (mean age of boys: 8.36, mean age of girls: 7.76) in the United States through the online Prolific platform were analyzed using path analysis and gender-based multi-group analysis. The results showed an inverse relationship between family stressors and functioning (β = −0.39, *p* < 0.05). COVID-19 family stress was negatively related to child physical HRQOL (β = −0.20, *p* < 0.05) but not psychosocial HRQOL. Family functioning showed a positive relation with child healthy food intake (β = 0.26, *p* < 0.05) and a negative relation with unhealthy diet consumption (β = −0.27, *p* < 0.05), while no significant associations were found with child physical activity and screen time. Family functioning was indirectly associated with both types of HRQOL through the child’s eating patterns. These relationships were more pronounced for girls. The findings point to a complex interplay between family stress and functioning, dietary habits, and the HRQOL of children during the COVID-19 pandemic, particularly concerning girls’ food intake and well-being.

## 1. Introduction

The emergence of the COVID-19 pandemic in late 2019 brought about widespread disruptions to daily life on a global scale. By January 2022, the United States had experienced the peak of its COVID-19 cases [1]. Subsequently, a gradual return to normalcy in American daily life became evident as the spread of COVID-19 subsided. This was characterized by the reopening of schools and workplaces, as well as the relaxation of COVID-19 prevention measures, although the pace of this process varied in different communities. However, the residual effects of the pandemic’s active phase persisted, particularly among young children [2], giving rise to unfavorable lifestyle behaviors. These included reduced levels of physical activity (PA), fewer healthy dietary choices [3], and increased screen time [4]. Also, previous studies have shown that children’s health-related quality of life (HRQOL) decreased throughout the phases of the pandemic compared to pre-pandemic levels [5,6].

Following the World Health Organization’s definition of health as “a complete state of physical, mental, and social well-being, not merely the absence of disease [7]”, the concept of HRQOL was developed to capture aspects of an individual’s subjective experience related to health, disease, disability, and impairment and the effects of medical treatment [8,9]. Furthermore, HRQOL encompasses the physical symptoms, functional status, and psychological and social impact of a health condition. HRQOL is closely linked to both physical and mental health, making it a powerful multidimensional tool to gauge overall health and well-being [10]. Thus, the importance of HRQOL is widely recognized by clinicians, researchers, and policy makers [11]. The need to understand HRQOL persisted due to the ongoing effects of the COVID-19 pandemic and its aftermath. By 2022, although vaccines and treatments may have been widely available [12], the pandemic’s lingering impacts on children’s physical, mental, and social well-being continued to be significant. Assessing children’s HRQOL remained essential in understanding how families were adapting to the “new normal [13]”, managing any persistent health or mental issues related to COVID-19, and addressing any long-term consequences on their quality of life.

### 1.1. COVID-19 Family Stress, Family Functioning, and Health-Related Quality of Life

The COVID-19 pandemic has had a significant impact on family dynamics, reshaping the ways in which families function, interact, and cope with unprecedented challenges [14]. Although this provided chances for family bonding, it also posed challenges in terms of finding a balance with the need for personal space and privacy. Lockdowns, school closures, and remote work arrangements placed a heavier burden on parents, resulting in increased parental responsibilities and stress compared to the pre-pandemic period [15]. Moreover, the anxiety and uncertainty associated with the pandemic took a toll on the mental health of family members [14]. The stressors of the pandemic, coupled with increased time spent together, could lead to conflicts within families, with possible lingering negative effects on the family environment, as well as on the well-being of individual members, including the children [16]. In addition to these psychosocial impacts, physical factors have been significantly highlighted, particularly due to lockdowns in the early stages and their lingering effects [17].

The family context is widely recognized as pivotal in shaping children’s acquisition of appropriate daily life habits, underscoring the significance of the family environment as a key determinant of children’s health and well-being [18]. Family functioning pertains to the operational dynamics of a family unit and the interplay among its members [19], encompassing communication patterns, roles, decision-making mechanisms, and emotional undercurrents [20]. Understanding family functioning involves examining interactions between family members, such as those between parents and children [21], and how these interactions influence overall relationships and effective communication methods. This plays a direct role in fostering positive health habits and influences the upbringing of children by their parents [22], impacting their academic performance [23], social behaviors [24], nutritional health [25], and emotional development [26]. Dysfunctional family dynamics can result in negative parental behaviors, hindering the development of essential skills in family members, particularly children [27].

Moreover, children’s lifestyle behaviors, such as dietary intake, PA, and screen time, have been found to be linked with HRQOL [28,29,30]. Specifically, healthy food intake has been related to better HRQOL in children [31], whereas snack food consumption has been related to poorer HRQOL [32]. Additionally, higher PA has been linked with better HRQOL [33], while screen time has shown the opposite trend [30]. Furthermore, when these were examined simultaneously in Chinese children and adolescents, it was observed that as the number of lifestyle risk behaviors increased, their reported HRQOL decreased [27]. This suggests the presence of unique and additive effects on children’s HRQOL among lifestyle behaviors. Thus, it is important to examine a range of lifestyle behaviors concurrently, to gain a comprehensive understanding of how they may be related to HRQOL and whether these lifestyle behaviors represent one mechanism by which family functioning is associated with children’s HRQOL.

### 1.2. Research Hypothesis

**Figure 1:**
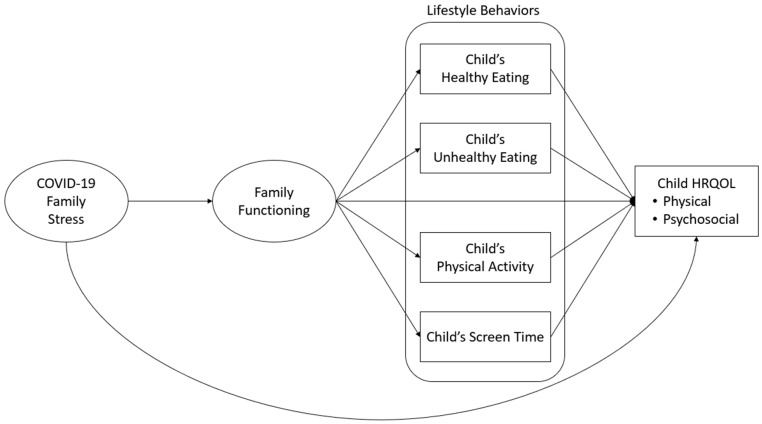
Conceptual model.

Considering the factors mentioned above, there is a need for an understanding of how COVID-19 family stress was related to child HRQOL as the impact of the pandemic lessened. Comprehending the combined effects of various lifestyle behaviors on HRQOL is central to developing holistic approaches to promote children’s overall health and quality of life, as lifestyle behaviors are malleable. Additionally, prior research has underscored gender differences in the incorporation of lifestyle behaviors and HRQOL, wherein boys spend more time with screens [5] and engage in more PA [34], while girls report lower HRQOL [35,36]. Establishing gender-specific normative values can enhance our understanding of gender differences and facilitate the provision of gender-sensitive healthcare [37,38,39]. Therefore, the present study aimed to examine the roles of family functioning and children’s lifestyle behaviors as potential mechanisms linking COVID-19 family stress and HRQOL according to children’s gender. We hypothesized specific relationships among these variables, as presented in Figure 1.

More specifically, the following hypotheses were tested:

(1) COVID-19 family stress is negatively related to family functioning and child physical and psychosocial HRQOL;
(2) Family functioning is positively associated with child healthy eating and PA and negatively associated with child unhealthy eating and screen time;
(3) Family functioning is positively related to child physical and psychosocial HRQOL;
(4) Child healthy food intake and PA are positively related to both child physical and psychosocial HRQOL;
(5) Child unhealthy diet and screen time are negatively related to child physical and psychosocial HRQOL;
(6) Children’s lifestyle behaviors convey indirect effects of family functioning on HRQOL.

## 2. Materials and Methods

### 2.1. Sample Recruitment and Data Collection Procedures

The data were collected through an online platform managed by Prolific Academic Ltd. [40], which has been created for the purpose of recruiting research participants [41]. The platform maintains a pool of approximately 37,000 potential research participants in the United States. For this study, Prolific selected a convenience sample of approximately 5500 participants residing in the United States, with the stipulations of participants being at least 18 years of age and having at least one child between ages 5 and 12. We selected this age group of children because they are in grade school before entering adolescence and still at an age when the direct influence of family functioning on lifestyle behaviors is likely to be present. The eligible participants could enroll on a first-come, first-served basis on 24 June 2022. Participants provided consent to participate in a survey and for the scientific publication of the findings, for which they were paid at a rate of USD 12/h. A total of 273 participants completed the survey. The collective data are registered at Mendeley Data [42], and the project was pre-registered at Open Science Framework Registries [43]. After deleting observations from participants whose child was not aged between 5 and 12, who failed two attention checks, and who completed the survey in an extremely short amount of time (<5 min), our maximum sample size became 223.

Numerous steps were taken to ensure the integrity of the data and methodological rigor. The survey was pre-tested manually and online with three adult volunteers to gain feedback on the clarity of the survey questions, the appropriateness of the items below, and the time required to complete the survey. Two attention checks were included; the first one stated “Pleased indicate your agreement with the statement below so that we know that you are paying attention. ‘I swim across the Atlantic Ocean to get to work every day’”, and the second one stated “The color test you are about to take part in is very simple. When you are asked for your favorite color, you must select ‘blue’. This is an attention check. Based on the text you read above, what color have you been asked to enter?”.

### 2.2. Study Measures

#### 2.2.1. COVID-19 Family Stress

COVID-19 family stress was assessed by the parent by completing the COVID-19 Family Stressor Scale (CoFaSS). CoFaSS was developed within a conceptual framework of COVID-19 and family resilience and comprises three scales for income stress, family stress, and chaos stress [44,45]. Each scale has shown acceptable internal consistency (i.e., income stress of five items, α = 0.75, family stress of seven items, α = 0.82, and chaos stress of four items, α = 0.68) [45]. Out of the three scales, we focused on family stress only for the current study. Each item was rated on a three-point scale to indicate the severity of experiencing each state since the outbreak of the COVID-19 pandemic, ranging from “not true” (1), to “somewhat true” (2) and “very true” (3). Examples of items include “children have become harder to manage”, “difficulty developing a new family and/or personal routine”, and “experienced increased altercations with family members”. Confirmatory factor analyses (CFAs) were conducted to create a latent variable. The internal consistency of a full family stress scale was α = 0.87 in the current sample.

#### 2.2.2. Family Functioning

Family functioning was assessed by the parent by completing the General Functioning subscale of the McMaster Family Assessment Device [20]. This consists of 12 items, six of which reflect healthy family functioning and six of which reflect unhealthy functioning. The responses for the negatively worded items were reverse-coded. Responses were provided on a four-point scale from “strongly agree” (1) to “strongly disagree” (4). Examples of items include “there are lots of bad feelings in the family”, “individuals are accepted for what we are”, and “in times of crisis, we can turn to each other for support”. CFAs were conducted to create a latent variable. Internal consistency of a full scale was α = 0.89 in the current sample.

#### 2.2.3. Child’s Dietary Intake

The child’s dietary habits were assessed using items adapted from the California Health Interview Survey Diet Screener [46]. This focuses on food categories commonly used in survey research to mark healthy and unhealthy dietary intakes [47,48]. The parent indicated the child’s intake for two food categories commonly associated with a healthy diet (fruits and vegetables) and four categories linked to unhealthy eating (juice, soda, sweets, and fast food). For each category, parents reported the number of servings that the child consumed the previous day, with response options ranging from “0” to “more than 8 servings”. The exception was fast food consumption, which was assessed over the past week. Parents determined servings based on their perception of a regular portion for their child. Healthy food intake was calculated from the total servings of fruits and vegetables, while unhealthy food intake was calculated from the total servings of juice, soda, sweets, and fast food.

#### 2.2.4. Child Physical Activity

The parent was asked to consider a 7-day period and report how many days their child did the following activities for more than 15 min during their free time (range 0–7): “strenuous exercise that heart beats rapidly, i.e., running, jogging, hockey, soccer, basketball, judo, roller skating, vigorous swimming, vigorous long-distance bicycling”; and “moderate exercise, i.e., fast walking, easy bicycling, easy swimming, dancing, etc.”. The total amount of PA was summed from responses to both strenuous and moderate activities.

#### 2.2.5. Child’s Screen Time

The parent was asked about their child’s screen time for leisure purposes separately for weekdays and weekends, using the following questions: “How many hours a day during the last 4 weeks did your child watch TV on a normal weekday/weekend day?” and “How many hours a day during the last 4 weeks did your child play console games or used a computer or a device/phone for free time activities on a normal weekday/weekend day?” Possible responses were in half-hour increments from “not at all” to “more than 6 h per day”. Total screen time was derived from the sum of indicated time spent on watching TV, computer/video games, and other screen devices.

#### 2.2.6. Child HRQOL

The Pediatric Quality of Life Inventory 4.0 (PedsQL) was completed by the parent to measure the HRQOL of the child [11]. This short form consists of 13 items, of which 8 address physical well-being (e.g., “walking more than one block”, “participating in sports activity or exercise”, “low energy level”) and 5 address psychosocial well-being (e.g., “feeling sad or blue”, “feeling angry”, “feeling afraid or scared”). Responses were made on a five-point scale (0 = never to 4 = always), with items then converted to a score out of 100 (0 = 100; 1 = 75; 2 = 50; 3 = 25; 4 = 0) by following manual procedures [11]. A mean score was calculated for the physical and psychological HRQOL scale [11]. The internal consistency was α = 0.75 for physical HRQOL and α = 0.81 for psychosocial HRQOL in the current sample.

#### 2.2.7. Race/Ethnicity

Parent indicated which ones of eight racial/ethnic categories described the child from the following: American Indian or Alaska Native, Asian, Black/African American, Caucasian/White, Hispanic/Latinx, Middle Eastern, Mixed and Multi-racial, or other race and ethnicity. Using the Census classification approach, the child was classified as Asian or Latinx if so indicated, regardless of other racial/ethnic indications. The child was categorized as “mixed and multi-racial” when there was more than one category checked.

### 2.3. Statistical Analysis

IBM SPSS Statistics 28 was used for descriptive statistics. Path analysis with Mplus statistical package was used to test overall model fit and hypothesized associations. The normality of all variables was checked first. Missing data were computed under maximum likelihood estimation [49]. In the process of identifying and confirming the appropriateness of a factor for a construct, it is essential to conduct CFA prior to performing path analysis [50]. CFA was employed to determine the factor loadings separately for COVID-19 family stress and family functioning. The compatibility of each factor was assessed to ensure alignment with the intended constructs. Three goodness-of-fit indices were examined to determine how well the model reproduced characteristics of the observed data: comparative fit index (CFI), Tucker–Lewis index (TLI), and root-mean-square error of approximation (RMSEA) [50]. After ensuring the adequate fit of the measurement models, structural models were estimated based on the hypothesized relationships displayed in Figure 1. CFI and TLI values above 0.95 are considered to indicate close fit, while values greater than 0.90 are considered to indicate acceptable fit [51]. RMSEA values of 0.05 or less indicate a close fit, whereas values of 0.08 or less indicate adequate fit [51,52]. Parents’ highest education level and the number of people living in the household were used as control variables to ensure that observed effects were not solely attributable to differences in parents’ socio-economic status or the number of children/adults in the same household. Finally, multi-group structural equation modeling (SEM) analysis was conducted to compare model fit and paths between boys and girls.

The characteristics of SEM, such as its flexibility in examining complex associations and its ability to handle various types of data, make it challenging to establish universal guidelines for sample size requirements [53]. Nevertheless, several rules of thumbs have been suggested, including (a) a minimum sample size of 100 or 200 [54,55], (b) 5 or 10 observations per estimated parameter [56,57], and (c) 10 cases per variable [58]. Given that the proposed research model (Figure 1) comprised 15 paths and 7 variables, including measurement models, a minimum of 190 participants was deemed satisfactory.

## 3. Results

### 3.1. Descriptive Statistics

Table 1 presents the characteristics of the study population, which consisted of 113 fathers and 109 mothers each reporting on a different child, providing responses that collectively included 119 boys and 102 girls. The majority of parents in this study had completed some college education or higher (fathers: 92.4%; mothers: 83.7%) as is typical for the Prolific pool. Additionally, a significant portion of the children in the sample were White, comprising 75.3% of the total. The descriptive statistics for the study variables are reported in Table 2. After checking the normality of all variables, square root transformation was applied to child unhealthy diet to achieve normality (Kurtosis 5.23 to Kurtosis 0.98) [59].

### 3.2. Path Analysis for the Total Sample

**Figure 2:**
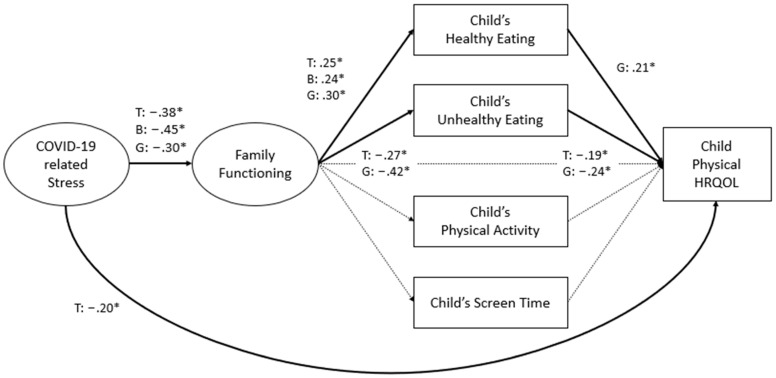
Path analysis results for physical HRQOL. Note: latent variables are depicted as ovals, while observed variables are represented by rectangles. Solid lines denote significant paths, while dashed lines represent insignificant paths. All paths controlled for parents’ highest education level and the number of people living in the household. T: total sample; B: boys; G: girls; * *p* < 0.05.

**Figure 3:**
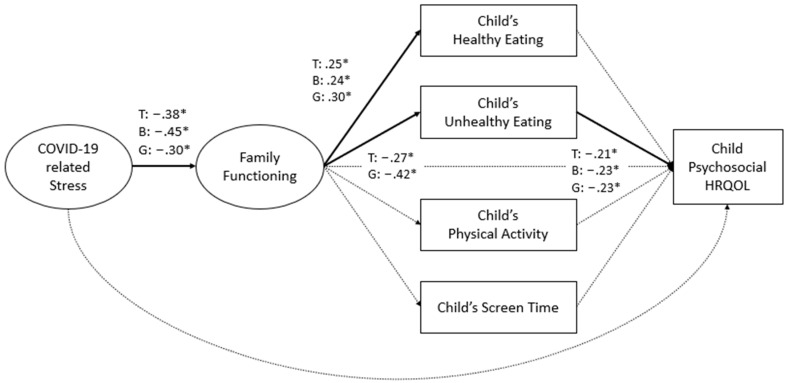
Path analysis results for psychosocial HRQOL. Note: Latent variables are depicted as ovals, while observed variables are represented by rectangles. Solid lines denote significant paths, while dashed lines represent insignificant paths. All paths were controlled for parents’ highest education level and the number of people living in the household. T: total sample; B: boys; G: girls; * *p* < 0.05.

After ensuring a satisfactory fit for the measurement models for COVID-19 family stress and family functioning, as shown in Table 3, an SEM path analysis of the model depicted in Figure 1 was conducted for the total group, for physical HRQOL and psychosocial HRQOL separately, as the final outcome variables. The results are shown in Figure 2 and Figure 3, respectively. Both models had a satisfactory fit to the data (physical HRQOL: CFI = 0.94; TLI = 0.92; RMSEA = 0.05, 90% CI [0.03, 0.08]; psychosocial HRQOL: CFI = 0.93; TFI = 0.90; RMSEA = 0.06, 90% CI [0.03, 0.08]).

There was a negative relationship between COVID-19 family stress and family functioning. COVID-19 family stress exhibited a negative association solely with child physical HRQOL and not with child psychosocial HRQOL. Family functioning showed a positive relation with child healthy food intake and a negative relation with unhealthy intake, while no significant associations were found with child PA and screen time. No significant direct paths were found between family functioning and child physical and psychosocial HRQOL. Among the four lifestyle behaviors examined, only child unhealthy food intake was related with HRQOL.

### 3.3. Multi-Group Path Analysis Results for Boys and Girls

Multi-group analysis for boys and girls was conducted separately for physical and psychosocial HRQOL, which resulted in a slightly reduced fit (physical HRQOL: CFI = 0.91; TFI = 0.88; RMSEA = 0.06, 90% CI [0.04, 0.08]; psychosocial HRQOL: CFI = 0.90; TFI = 0.86; RMSEA = 0.07, 90% CI [0.04, 0.08]). This is typically due to a smaller sample size for sub-groups compared with the total sample. However, based on the acceptable fit from the total sample and acceptable RMSEA results in the multi-group analysis, the model fits the data sufficiently to be interpreted.

As shown in Figure 2 and Figure 3, there was a negative association between COVID-19 family stress and family functioning for boys and girls, but not between COVID-19 family stress and child HRQOL. There were significant pathways related to child food intake, but not PA and screen time. Specifically, family functioning was positively associated with healthy food intake for boys and girls. Moreover, family functioning was negatively related only to girls’ unhealthy food consumption. There was no direct association between family functioning and child HRQOL. Girls’ unhealthy food consumption was linked to lower physical HRQOL, and for both boys and girls, unhealthy food intake was related to lower psychosocial HRQOL.

### 3.4. Indirect Pathways

We conducted further analysis related to the indirect paths between family functioning and child physical and psychosocial HRQOL through child unhealthy food consumption, which was the only lifestyle behavior that was found to be significantly associated with HRQOL (see above). As shown in Table 4, in the total group analysis, indirect effects were observed between family functioning and both child physical and child psychosocial HRQOL, through child unhealthy food intake. Likewise, within the gender-specific analysis for girls, there was a significant indirect effect between family functioning and child psychosocial HRQOL through child unhealthy food intake. Because there was no relationship between unhealthy intake and HRQOL for boys (see above), there could be no indirect effect to test.

## 4. Discussion

This study delved into the links between societal changes incurred during the later stages of the COVID-19 pandemic and family dynamics, revealing an expected inverse relationships between family stressors and family functioning. Furthermore, the results highlighted the role of dietary habits in mediating between family functioning and HRQOL. Specifically, family functioning exhibited connections with a higher healthy food intake and a lower unhealthy food intake in children. In contrast, there were no associations found for the other lifestyle behaviors of child PA and screen time with either family functioning or child HRQOL. The current study underscored the associations of child unhealthy food consumption with both lower physical and psychosocial HRQOL. Moreover, family functioning was indirectly associated with both child physical and psychosocial HRQOL via the child’s unhealthy eating patterns, consistent with an interpretation of the mediation effect. Notably, we found gender-specific associations, wherein for girls only family functioning was related to unhealthy food intake, which, in turn, was associated with their physical HRQOL.

### 4.1. COVID-19 Family Stress and Family Functioning

The adverse association between COVID-19 family stress and family functioning is noteworthy because of the timing of this data collection. Despite being collected after the lifting of shelter-in-place and lockdown measures throughout the United States in 2022, the results presented a significant negative relationship between stressful experiences related to COVID-19 and poorer family functioning. The social disruptions stemming from the pandemic have been reported to induce heightened psychosocial distress across populations, including parents and children [15,60,61]. This increase, on average, likely affected the quality of relationships both between parents and among parents and their children [44]. Consistent with family stress theory, caregivers confronted with significantly increased stress may have found their mental and emotional resources depleted, rendering the task of positive leadership within the family challenging, if not overwhelming [62]. In a partially similar context, amidst the Farm Crisis of the 1980s, rural families encountered heightened societal pressure that drained their resources, impacted mental well-being, and hindered their ability to foster constructive guidance within the family [62]. Families may encounter the detrimental impacts of stress originating from various sources, such as economic downturns, military deployment, and company layoffs. This, of course, extends to the challenges posed by the COVID-19 pandemic. There is a clear need for programs and policies that provide support to families during periods of stress.

### 4.2. COVID-19 Family Stress and Child Physical HRQOL

Our study revealed an association between COVID-19 family stress and reduced physical HRQOL in children, whereas no such direct connection was found with children’s psychosocial HRQOL. We postulated that children might have been less psychosocially affected by COVID-19 family stress due to their early stage of development and perhaps their limited mental maturity. Alternatively, these young children might have shown resilience, bouncing back from the negative psychosocial effects during the active early pandemic phase, as some aspects of their lives returned to being in a normal range. Furthermore, the pandemic restrictions might have limited their PA opportunities during a critical developmental phase, potentially hindering the formation of a solid foundation for future PA habits. For instance, reduced access to sport facilities, park closures, and limitations on outdoor play due to safety concerns may have led to decreased PA levels among children [63]. Additionally, the pandemic stressors might have depleted parents’ energy or reduced their willingness to engage in sports activities with their children or provide necessary logistical support for their physical HRQOL. These findings accentuate the importance of considering how pandemic-related stressors are related to various aspects of children’s health and the need to support PA opportunities for children’s overall well-being.

### 4.3. Family Functioning and Child Food Intake

Our simultaneous examination of lifestyle behaviors emphasized the significant association between family functioning and dietary habits. In addition, one possible indirect effect emerged between family functioning and child psychosocial HRQOL through unhealthy food intake. Effective family dynamics, characterized by well-defined roles, open communication, and managed emotional expressions, generally enable families to fulfill their responsibilities while fostering individual growth and well-being [64]. Conversely, poor family function may contribute to the development of unhealthy habits, such as unhealthy eating, as indicated by our findings. Family conflicts might prompt parents to exhibit maladaptive stress and emotional eating behaviors, which children may mimic [65]. Additionally, parents may resort to comfort foods as a means of emotional regulation and apologetic compensation following conflicts [66]. Consequently, family functioning has shown strong associations with various health-related behaviors and outcomes in children, encompassing physical, social, and mental well-being, as well as influencing risk-taking behaviors [67]. Overall, family functioning influences children’s lifestyle behaviors by shaping the transmission of health habits through guiding parental approaches to their children [68]. Therefore, there is a need for efforts to elucidate the underlying mechanisms for leveraging family functioning that would enable families to employ effective and practical tools to enhance positive family dynamics in their daily lives [25].

### 4.4. Unhealthy Diets in Children and Their Psychosocial HRQOL

Our finding of an association between increased consumption of unhealthy foods, such as more fast food, sweets, and candies, and lower psychosocial HRQOL aligned with the existing research [32,69]. Unhealthy dietary habits have been associated with a higher prevalence of mental health issues in children, including unhappiness, depression, and perceived stress [70]. Biological factors associated with unhealthy eating may underlie these associations. For instance, diets high in processed foods and sugar and lacking essential nutrients can adversely affect children’s brain function and neurotransmitter production, potentially affecting mood regulation [71]. Furthermore, unhealthy diets might lead to inflammation in the body, disrupting the gut microbiome and altering the gut-brain balance, which can impact mood and mental health [72]. Similar connections have been observed in older children, in that a higher fast food and sugary beverage intake was linked to lower psychosocial HRQOL [32,69]. These findings point to the value of finding effective ways of reducing unhealthy intake in childhood as early as possible. Not only could physical health be improved, but there could also be broader effects on the psychosocial well-being of children.

### 4.5. Child Gender Differences

The multi-group analysis based on the children’s gender revealed more pronounced associations between family functioning and HRQOL through food intake among girls. Specifically, girls exhibited a link between healthy food intake and better physical HRQOL and between unhealthy eating and worse psychosocial HRQOL. There were no associations specific to boys. Previous research has underlined gender disparities in food choices and dietary patterns, likely influenced by societal expectation and stereotypes transmitted through parental, peer, and media influences [73]. The findings suggest that societal influences, biological differences, and gender-specific factors likely contribute to these disparities in how girls and boys respond to dietary habits within the family context. This supports the consideration of a gender-specific approach when addressing children’s dietary behaviors and their impact on overall well-being.

### 4.6. Limitations

First, the cross-sectional observational design employed in this research precludes the determination of causation. Whereas we tested a hypothesized model of relationships grounded in theory and prior research, the direction of effects implied therein are offered as one interpretation among several. Furthermore, the sample is primarily comprised of White individuals sourced from an online platform, potentially limiting the generalizability of the results. It is important to note that the COVID-19 pandemic disproportionately affected Black, Latinx, and Native American communities more severely [74,75], highlighting potential limitations in generalizing these findings to other racial and ethnic groups. Also, due to the young age of the children in our sample and this research being conducted entirely online, only the primary caregiver completed the questionnaires. It is desirable in family research to obtain reports from child family members when feasible. Lastly, family functioning during the pandemic likely reflects, to a large extent, their functioning before the pandemic, which could not be addressed in this research [76]. Future research could be enhanced by integrating pre-pandemic data to provide a more comprehensive context for interpreting the findings and understanding the unique impact of the pandemic on the variables studied.

## 5. Conclusions

Overall, this investigation underscores the intricate relationships between family stress, family functioning, and dietary habits, and their collective impact on the HRQOL of children amidst the COVID-19 pandemic. It sheds light on gender-specific nuances, notably regarding girls’ food intake and well-being. This study underscores the pivotal role of family functioning in bolstering HRQOL. Additionally, the addressing of unhealthy food consumption emerges as a potential avenue for enhancing children’s well-being.

## Figures and Tables

**Table 1:** Sample demographic information.

Variable	
Parent’s gender	%
Father (*N* = 113)	50.9
Mother (*N* = 109)	49.1
Parent’s age	M years
Father	38.6 (SD = 7.1)
Mother	39.7 (SD = 8.0)
Respondent’s relationship to the child	%
Biological mother	43.4
Biological father	48.7
Adoptive mother	1.8
Adoptive father	0.4
Foster father	0.9
Stepfather	0.9
Grandparent	3.1
Parent’s partner, boyfriend, or girlfriend	0.4
Other relative (brother, sister, uncle, aunt, etc.)	0.4
Father’s educational level	%
Some high school but did not graduate	0.8
High school graduate and GED	6.7
Some college, or 2-year degree (AA or AS)	19.3
4-year college graduate (BA, BB, or BS)	52.9
More than a 4-year college degree (Ph.D., MD, etc.)	20.2
Mother’s educational level	%
8th grade or less	1.8
Some high school but did not graduate	1.8
High school graduate and GED	12.6
Some college, or 2-year degree (AA or AS)	22.5
4-year college graduate (BA, BB, or BS)	41.4
More than a 4-year college degree (Ph.D., MD, etc.)	19.8
Child’s gender	%
Boy (*N* = 119)	53.6
Girl (*N* = 102)	45.9
Child’s age	M years
Boy	8.4 (SD = 2.5)
Girl	7.8 (SD = 2.2)
Child’s race	%
Asian	1.3
Black	5.7
Latinx	2.2
White	75.3
Other	15.4

**Table 2:** Descriptive statistics for study variables.

Variables	Scale	Total	Boys	Girls
		M (SD)	M (SD)	M (SD)
COVID-19 Family Stress				
Experienced increased altercations with family members.	1–3	1.26 (0.50)	1.20 (0.49)	1.27 (0.51)
Experienced increased emotional withdrawal from family members.		1.39 (0.62)	1.35 (0.61)	1.43 (0.64)
Children have become harder to manage.		1.44 (0.64)	1.45 (0.61)	1.43 (0.67)
More relationship conflicts with my partner.		1.27 (0.52)	1.24 (0.47)	1.31 (0.58)
Family Functioning				
In times of crisis, we can turn to each other for support.	1–4	3.48 (0.60)	3.54 (0.56)	3.41 (0.64)
We can express feelings to each other.		3.35 (0.60)	3.37 (0.60)	3.33 (0.59)
We are able to make decisions about how to solve problems.		3.36 (0.66)	3.43 (0.63)	3.29 (0.67)
We confide in each other.		3.60 (0.62)	3.68 (0.56)	3.51 (0.66)
Child Healthy Food Intake	1–10	3.68 (2.02)	3.55 (2.03)	3.91 (2.03)
Child Unhealthy Food Intake	1–20	4.03 (3.11)	2.99 (2.37)	3.87 (3.47)
Child Physical Activity	0–14	6.81 (4.10)	7.18 (4.22)	6.57 (4.07)
Child Screentime	0–28	9.36 (5.32)	9.91 (4.91)	8.60 (5.55)
Child Physical HRQOL	0–100	88.81 (11.71)	89.23 (12.01)	88.34 (11.40)
Child Psychosocial HRQOL		82.15 (15.78)	82.64 (15.65)	81.59 (15.98)

**Table 3:** Factor loadings for latent variables and model fit for the measurement models.

Items	Factor Loadings	CFI	TLI	RMSEA
COVID-19 Family Stress				
Experienced increased altercations with family members.	0.83	0.99	0.97	0.08 90% CI: 0.00, 0.17
Experienced increased emotional withdrawal from family members.	0.70			
Children have become harder to manage.	0.61			
More relationship conflicts with my partner (if I am in a relationship).	0.72			
Family Functioning				
In times of crisis, we can turn to each other for support.	0.77	1.00	1.00	0.00 90% CI: 0.00, 0.13
We are able to make decisions about how to solve problems	0.86			
We can express feelings to each other.	0.72			
We confide in each other.	0.82			

Note. CFI: comparative fit index; TLI: Tucker–Lewis index; RMSEA: root-mean-square error of approximation.

**Table 4:** Indirect effects.

Indirect Effect	Estimate (β)	SE	Bootstrap 95% CI
Total Group			
Family functioning →	0.05	0.01	0.02, 0.10
Child unhealthy food intake →			
Child physical HRQOL			
Family functioning →	0.06	0.02	0.02, 0.10
Child unhealthy food intake →			
Child psychosocial HRQOL			
Girls			
Family functioning →	0.10	0.05	0.02, 0.20
Child unhealthy food intake →			
Child psychosocial HRQOL			

Note. CI = confidence interval.

## Data Availability

The data used in this study are available and can be found in Mendeley data, V1, https://doi.org/10.17632/2kf2tzdd7y.1 (accessed on 7 December 2023).
